# Case Report: High-Dose Ferric Carboxymaltose as an Antianaemic Agent to Avoid Haemotransfusions after Total Hip Replacement

**DOI:** 10.3390/medicina60081274

**Published:** 2024-08-07

**Authors:** Maiya Konkayeva, Assiya Kadralinova, Nazerke Zhanarystan, Nurlan Akhatov, Aidos Konkayev

**Affiliations:** 1 Department of Anesthesiology and Intensive Care, National Scientific Center of Traumatology and Orthopaedics Named after Academician N.D. Batpenov, Astana 010000, Kazakhstan; ysaimnida@mail.ru (A.K.); konkaev19@gmail.com (A.K.); 2 Department of Anesthesiology and Intensive Care, Astana Medical University, Astana 010000, Kazakhstan; zhanarystan97@mail.ru (N.Z.); nurlandiana@mail.ru (N.A.)

**Keywords:** ferric carboxymaltose, high dose, preoperative anaemia, iron supplements, total hip replacement, case report

## Abstract

This article highlights a case of high-dose ferric carboxymaltose (Ferinject^®^) for the treatment of perioperative iron deficiency anaemia in a 39-year-old patient with dysplastic coxarthrosis. The patient was admitted routinely for a total hip replacement of the left hip joint. She had been suffering from pain, lameness, and restriction of movement in her left hip joint for the past several years. The patient was admitted with initial iron deficiency anaemia of a medium severity (Hgb—96.5 g/L, RBC—3.97 × 10^12^/L). Laboratory tests were taken to determine the iron deficiency, and transfusion readiness was submitted. The patient received ferric carboxymaltose infusion before surgery. The intraoperative blood loss was—100 mL with an operation duration of 50 min. On the first postoperative day, haemoglobin decreased to 86 g/L. No haemoglobin decrease was observed in the postoperative period, and 92 g/L was the amount of haemoglobin at the time of hospital discharge. The optimal dose for the treatment of perioperative anaemia has not been established; some studies recommend ferric carboxymaltose at a dose of 15 to 20 mg/kg and a maximum of 1000 mg once on the first day after surgery. The uniqueness of this case report is that a high dose of ferric carboxymaltose (1340 mg) during the preoperative period was applied. No side effects such as hypophosphatemia were reported. We believe that, in this clinical case, the patient managed to avoid large intraoperative blood loss and transfusions by using high doses of ferric carboxymaltose.

## 1. Introduction

Large joint replacement procedures are becoming more commonplace, favourable experiences in this field and are expanding. Anaesthetic and pharmacological intraoperative support quality are rising, in addition to implanted material quality and operator expertise. This advancement in orthopaedics helps patients with various musculoskeletal disorders live better lives by minimising their disabilities. Nevertheless, there is a risk of developing serious, postoperative, infectious complications such as the development of periprosthetic joint infections, which worsen the patient’s prognosis and require additional joint replacement revisions.

It is believed that perioperative anaemia, including latent iron deficiency anaemia (suppressing the cellular mechanisms of immunity), is considered an independent and major factor in the development of periprosthetic infection. Preoperative anaemia can reduce the level of C3b receptor content on the erythrocyte surface, reduce the immune functions of the body, increase the risk of septic complications, prolong postoperative rehabilitation time, and lead to periprosthetic infection [1,2].

The risk of acute renal injury and postoperative infection is also higher in patients with preoperative anaemia. In addition, some studies show that anaemia more strongly correlates with mortality than it does with morbidity due to cardiovascular and thromboembolic complications [3].

On the other hand, it has been demonstrated that allogeneic blood transfusions are a separate risk factor for higher unfavourable outcomes, including extended hospital stays and the emergence of possible infectious complications after surgery.

Specifically, preoperative anaemia has been associated with a growth in the number of blood transfusions conducted. Although several preoperative haemocorrection options have been proposed, there are no clear recommendations on how to prepare patients with preoperative anaemia for surgery in orthopaedics.

According to some studies, it is recommended to use a dosage of ferric carboxymaltose from 15 to 20 mg/kg body weight and a maximum of 1 g once on the first postoperative day [4,5,6]. We believe that personalised dosing regimens should be used as an insufficient dosage for a given body weight may not cover the body’s needs.

This study aimed to determine the effect of an individually adjusted, high-dose ferric carboxymaltose infusion given preoperatively to preclude intraoperative blood loss and the need for transfusion.

In this case study, we used an individualised dosage of 20 mg/kg for the patient’s ideal body weight and applied a maximum of 1340 mg of ferric carboxymaltose intravenously in a dilution with 400 mL of 0.9% sodium chloride solution for one hour once before surgery.

## 2. Case Presentation

### 2.1. Patient Information

A 39-year-old female patient was admitted for a planned unilateral hip replacement surgery with complaints of severe pain and restriction of movement in the left hip joint. She considers herself as being in pain since childhood, when the pain syndrome first appeared, and associates it with a congenital dislocation of the femoral head. In 2012, she underwent total hip replacement of the right hip joint due to the failure of conservative treatment. A revision hip joint endoprosthesis procedure was carried out in June of 2017. There were no incidents during the recovery time. The patient indicated that the pain in their left hip joint had increased over the past five years. After conservative therapy proved fruitless, she was admitted for a total hip replacement of her left hip joint. The patient’s only medical conditions are iron deficiency anaemia, excessive body weight, and congenital pathology, which have left her disabled. Her gynaecological anamnesis, allergies, and heredity are unremarkable.

### 2.2. Clinical Findings

The patient’s objective status was unremarkable. She weighed 93 kg with a height of 177 cm; the ideal body weight according to Broca’s formula was 67 kg. The body mass index was 29.7 kg/m^2^, which indicated that the patient was overweight.

The skin and visible mucous membranes were physiologically coloured, without rashes and peripheral oedema. Her breathing was independent, adequate, auscultatory, and percussive without pathology and with a respiratory rate of 18 breaths per minute. Her hemodynamic parameters were stable, and at the time of the examination, her normotension with arterial pressure was 120/80 mmHg, and her heart rate was 72 beats per minute. The organs of the digestive and urogenital systems were at objective examination without any deviations from the norm. On examination of the musculoskeletal system, the patient had pelvic distortion, shortening of the right lower limb, up to 2.0 cm. She also had a postoperative scar of the right hip joint without signs of inflammation. The volume of movement of the left hip joint was sharply limited and painful, while movements of the right hip joint were limited but painless. There were no neurological or vascular disorders in the distal limbs. The patient moved independently while limping on the right lower limb.

### 2.3. Timeline

**Figure 1:**
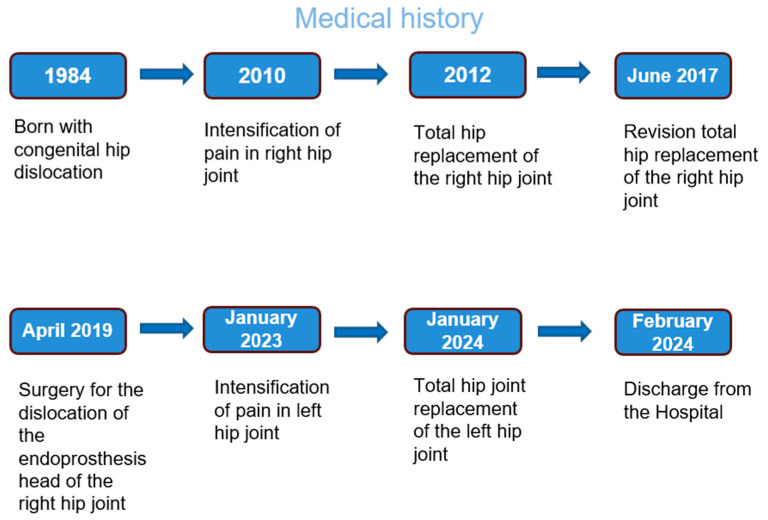
Patient’s medical history.

Figure 1 illustrates the chronological sequence of the patient’s medical history.

### 2.4. Diagnostic Assessment

The patient’s laboratory results upon admission revealed iron deficiency anaemia, with an erythrocyte count of 3.97 × 10^12^/L and a haemoglobin amount of 96.5 g/L. The colour index was—0.73, indicating the presence of hypochromic anaemia. The patient was additionally investigated for an iron deficiency before surgery. On admission of the biochemical blood test, iron was—5.1 mmol/L, transferrin—264 mg/dL, and ferritin—20.0 µg/L. Further, a high-dose infusion of ferric carboxymaltose at a dosage of 1340 mg was given immediately after the iron deficiency was detected.

The analyses were taken again the next day before the intervention. On that day, after the ferric carboxymaltose infusion, the levels of iron, transferrin, and ferritin rose to 28.6 mmol/L, 276.0 mg/dL, and 74.6 μg/L, respectively, according to the analyses. Following surgery, ferritin increased to 557 µg/L, transferrin reduced to 136.0 mg/dL, and iron levels dropped to 13.4 mmol/L.

Initially, the patient’s leukocytes were 5.62 × 10^9^/L, but after the operation, we observed an increase in leukocytes up to 10.10 × 10^9^/L, which we attributed to the surgical intervention. At her hospital discharge, the leukocytes returned to normal and amounted to 6.07 × 10^9^/L. The patient received antibiotics (cefazolin) as per institutional protocols meant to prevent infectious complications.

The inability to determine blood phosphate levels due to a lack of reagents was considered a diagnostic challenge. However, no clinical signs of hypophosphatemia, such as muscle weakness, seizures, heart failure, and other side effects described in the literature from the use of a high-dose infusion of ferric carboxymaltose, were detected. In our opinion, hypophosphatemia can be avoided by the use of oral or intravenous phosphate.

Both before and after surgery, coagulation measurements and other biochemical markers, such as urine tests, were within normal ranges. After anticoagulation therapy, the coagulation parameters were maintained at target normocoagulation values (prothrombin time—13.3 s, prothrombin time ratio—0.82%, INR—1.03, fibrinogen—6.32 g/L), except for the fibrinogen level.

Before surgery, the patient underwent routine screening for infections, including viral hepatitis B and C, HIV infection, and Wasserman reaction. All results were negative.

Among the instrumental methods of investigation, a check-up of the cardiovascular system electrocardiogram and echocardiogram was performed. The study showed no pathological changes. The respiratory system was also investigated, and fluorography was performed on an outpatient basis, where no abnormalities were detected. During a fibrogastroduodenoscopy, superficial gastritis was discovered. The veins of the lower limbs were also examined by ultrasound method; the veins were passable, and there was no thrombosis. The patient was then consulted by a general practitioner and admitted to surgery.

According to the above, the patient was diagnosed with bilateral dysplastic coxarthrosis of the third degree. Crowe type I dysplasia on the left and right hip joints. The condition after total hip replacement of the right hip joint in 2012. The condition after revision endoprosthesis of the acetabular component from 2017. Concomitant pathology included iron deficiency anaemia of medium severity and superficial gastritis out of the exacerbation stage.

### 2.5. Therapeutic Intervention

After obtaining informed consent and determining the patient’s iron deficiency through testing, the patient received a high-dose infusion of ferric carboxymaltose on the first day of the preoperative period at a dosage of 20 mg/kg of ideal body weight for antianaemic purposes. It was diluted once intravenously over an hour using 400.0 mL of 0.9% of sodium chloride solution or 1340 mg of the medication.

Further, in the postoperative period, antibacterial therapy was continued using cefazolin 1000 mg intravenously twice a day. The patient also received postoperative analgesia on the first day after surgery in the form of narcotic analgesics: trimeperidin 20 mg intramuscularly three times every 5 h. Further analgesia was maintained with a combination of non-steroidal, anti-inflammatory drugs: ketoprofen 100 mg intramuscularly twice a day for seven days.

To prevent thromboembolic problems in the postoperative phase, the patient was additionally administered low-molecular-weight heparin, namely calcium nadroparin 2850 IU/0.3 mL, subcutaneously once within three days following surgery. After that, dabigatran etexilate 220 mg was administered once daily for four days to complete the therapy. Additionally, the patient was given proton pump inhibitor medications (omeprazole 20 mg once a day) for seven days following the procedure to prevent stress ulcers. There were no indications of a need for blood transfusions.

Additionally, the patient received №5 in mechanotherapy and kinesiotherapy as part of their rehabilitation therapy.

### 2.6. Surgical Treatment

On the 3rd day of hospitalisation, the patient underwent total hip replacement of the left hip joint under spinal anaesthesia. The duration of the operation was 50 min. Intraoperative blood loss was 100 mL, and sedation therapy with dexmedetomidine at a dosage of 1 mcg/kg/h was used during the operation. In order to prevent blood loss, 1000 mg of tranexamic acid was intravenously used. Infusion therapy in a volume of 1500 mL of 0.9% sodium chloride solution was carried out, and prophylactic antibacterial therapy in the form of antibiotics of cephalosporin series of first-generation cefazolin 1000 mg was also intravenously applied. Nausea and bradycardia up to 50 beats/min were observed intraoperatively. Symptoms were controlled with an administration of one dose of 4 mg IV ondansetron and 0.5 mg IV atropine, respectively.

### 2.7. Follow-Up and Outcomes

Upon her discharge, the patient’s condition improved to a state where she could continue rehabilitation after surgery on an outpatient basis. The patient was discharged on the 9th day of hospitalisation with haemoglobin 92 g/L and a red blood cell count of 3.4 × 10^12^/L. Also, the colour index increased to 0.81. No intrahospital haemotransfusions were performed due to a lack of indication, and likewise, no side effects described in the literature such as hypophosphatemia or muscle weakness were reported.

A review of the patient on postoperative days 28 and 90 found that rehabilitation was successful, with no infectious complications or other adverse events. There was high adherence to these recommendations for the treatment of iron deficiency anaemia in the postoperative period. After a week, the patient received a repeat infusion of ferric carboxymaltose at a dose of 1000 mg IV. On day 90, haemoglobin was 118 g/L as a result of self-examination.

## 3. Discussion

There is a correlation between perioperative anaemia and a higher risk of red blood cell transfusion and increased morbidity and mortality after surgery. Anaemia raises a patient’s risk of death from respiratory (hypoxia and respiratory failure), cardiac (myocardial infarction, congestive heart failure, and cardiac arrest), septic, multi-organ failure, and haemorrhagic diseases when compared to non-anaemic patients. The most effective strategy for perioperative anaemia treatment is still up for debate [7,8,9].

Preoperative anaemia can have a variety of causes, but iron deficiency anaemia accounts for nearly two-thirds of anaemia in patients undergoing elective surgery. According to the International Consensus Conference on Anaemia Management in Surgical Patients, it is essential to diagnose iron deficiency, even in those patients with inflammation-related anaemia (also known as anaemia of chronic illness) [10].

Iron deficiency not only leads to anaemia; it also has other consequences. This is because iron is present in many important cellular proteins such as myoglobin and cytochromes. Therefore, even with normal haemoglobin values, iron deficiency can be found and associated with fatigue that can be managed by iron supplementation [11,12].

M. Munoz et al. concluded in their observational study that short-term perioperative intravenous iron administration, with or without erythropoietin preparations in patients undergoing major orthopaedic surgery, is associated with a reduction in the frequency of allogeneic haemotransfusions and length of hospital stays, without increasing postoperative morbidity and mortality [13].

A. Elhenawy et al. in their study support the idea of using intravenous iron supplementation before surgery to lower the risk of an allogeneic blood transfusion and provide a modest elevation to the haemoglobin concentration [14].

An observational study by S. Vikrant et al. assessing the use of high-dose iron in kidney disease found that a high dose of 1000 mg is well tolerated and safe [15]. In our study, an individual dose of 1340 mg was selected. The instruction booklet for ferric carboxymaltose (Ferinject) indicates the calculation of iron deficiency based on the patient’s haemoglobin, iron level, and weight. As a result, the patient’s haemoglobin was below 100 g/L (Hgb—96.5 g/L), and iron was less than 6.2 mmol/L (iron—5.1 mmol/L). With a weight above 70 kg (weight—93 kg), the iron deficiency amounted to 2000 mg. Since the recommended maximum dosage of ferric carboxymaltose is 1000 mg, an individualised dose of 20 mg/kg per a patient’s ideal body weight was selected, resulting in a total of 1340 mg. The patient was advised to repeat the ferric carboxymaltose infusion after one week while monitoring the analyses.

Ferric carboxymaltose, an intravenous iron formulation, allows for the potential of giving a lot of iron with one infusion. Because of this, anaemia is corrected more quickly, and the formulations are better tolerated than oral iron formulations. However, because these formulations strongly elevate the hormone-active fibroblast growth factor—23, which accelerates renal phosphate excretion, they may cause hypophosphatemia; however, the risk of these hypersensitivity reactions can be reduced by choosing a slow infusion rate [16,17].

According to a meta-analysis by B. Schaefer and co-authors, ferric carboxymaltose is associated with a high risk of developing hypophosphatemia that does not resolve for at least 3 months in a significant proportion of patients. More severe iron deficiency and normal renal function are risk factors for the development of hypophosphatemia [18].

As there is substantial evidence on the risk of hypophosphatemia with ferric carboxymaltose, ferric carboxymaltose infusion should be used with caution in patients with baseline osteoporotic changes during orthopaedic surgery. But, in this case report, no clinical signs of hypophosphatemia were recorded, probably due to the young age of the patient. The lack of laboratory monitoring of phosphate is also considered a limitation of this study.

A strength of this study is that very few data are presented on the use of high-dose ferric carboxymaltose in orthopaedic patients with initial preoperative iron deficiency anaemia. In a retrospective study by Kim SK et al., 150 orthopaedic patients received a maximum 1 g ferric carboxymaltose infusion on the first postoperative day. The authors of the study concluded that postoperative IV-FCM reduced the need for blood transfusion without influencing the clinical outcomes of the patients [19].

Since ferric carboxymaltose can lead to hypophosphatemia and osteomalacia, its use in patients with bone pathology, particularly osteoporosis, is questionable. However, in the Bernabeu-Wittel M study, the authors concluded that ferric carboxymaltose is safe and effective in patients with osteoporotic hip fractures [20,21,22].

## 4. Conclusions

In summary, the preoperative administration of high-dose ferric carboxymaltose probably prevented significant intraoperative blood loss and haemotransfusions in this case. However, additional research in this area is required.

### Patient Perspective

The patient experienced no adverse or undesirable reactions both during and after the infusion of ferric carboxymaltose. The patient’s well-being was reported as unchanged.

## Data Availability

The data presented in this study are available on request from the corresponding author. The data are not publicly available to ensure the confidentiality of the patient’s personal information.

## References

[1] Zhang F.Q., Yang Y.Z., Li P.F., Ma G.R., Zhang A.R., Zhang H., Guo H.Z. (2024). Impact of preoperative anemia on patients undergoing total joint replacement of lower extremity: A systematic review and meta-analysis. J. Orthop. Surg. Res..

[2] Wang W., Liu J., Zhou S.H., Li S.G., Qiao Y.J., Liu J., Zhen P. (2018). Periprosthetic joint infection after artificial joint replacement and preoperative anaemia. Zhongguo Gu Shang.

[3] Fowler A.J., Ahmad T., Phull M.K., Allard S., Gillies M.A., Pearse R.M. (2015). Meta-analysis of the association between preoperative anaemia and mortality after surgery. Br. J. Surg..

[4] Houry M., Tohme J., Sleilaty G., Jabbour K., Gebrael W.B., Jebara V., Madi-Jebara S. (2023). Effects of ferric carboxymaltose on haemoglobin level after cardiac surgery: A randomized controlled trial. Anaesth. Crit. Care Pain Med..

[5] Khalafallah A., Yan C., Al-Badri R., Robinson E., E Kirkby B., Ingram E., Gray Z., Khelgi V., Robertson I.K., Kirkby B.P. (2016). Intravenous ferric carboxymaltose versus standard care in the management of postoperative anaemia: A prospective, open-label, randomised controlled trial. Lancet Haematol..

[6] Spahn D.R., Schoenrath F., Spahn G.H., Seifert B., Stein P., Theusinger O.M., Kaserer A., Hegemann I., Hofmann A., Maisano F. (2019). Effect of ultra-short-term treatment of patients with iron deficiency or anaemia undergoing cardiac surgery: A prospective randomised trial. Lancet.

[7] Diress G.M., Ayele G. (2023). Prevalence and risk factors of preoperative anemia in patients undergoing elective orthopedic procedures in Northwest Ethiopia: A multicenter prospective observational cohort study. Patient Saf. Surg..

[8] Muñoz M., Gómez-Ramírez S., Campos A., Ruiz J., Liumbruno G.M. (2015). Pre-operative anaemia: Prevalence, consequences and approaches to management. Blood Transfus..

[9] Muñoz M., Acheson A.G., Auerbach M., Besser M., Habler O., Kehlet H., Liumbruno G.M., Lasocki S., Meybohm P., Baikady R.R. (2017). International consensus statement on the peri-operative management of anaemia and iron deficiency. Anaesthesia.

[10] Shander A., Corwin H.L., Meier J., Auerbach M., Bisbe E., Blitz J., Erhard J., Faraoni D., Farmer S.D., Frank S.M. (2023). Recommendations from the International Consensus Conference on Anaemia Management in Surgical Patients (ICCAMS). Ann Surg..

[11] DeLoughery T.G. (2014). Microcytic anaemia. N. Engl. J. Med..

[12] Camaschella C. (2015). Iron-Deficiency Anaemia. N. Engl. J. Med..

[13] Muñoz M., Gómez-Ramírez S., Cuenca J., García-Erce J.A., Iglesias-Aparicio D., Haman-Alcober S., Ariza D., Naveira E. (2014). Very-short-term perioperative intravenous iron administration and postoperative outcome in major orthopedic surgery: A pooled analysis of observational data from 2547 patients. Transfusion.

[14] Elhenawy A.M., Meyer S.R., Bagshaw S.M., MacArthur R.G., Carroll L.J. (2021). Role of preoperative intravenous iron therapy to correct anaemia before major surgery: A systematic review and meta-analysis. Syst. Rev..

[15] Vikrant S., Parashar A. (2015). The safety and efficacy of high dose ferric carboxymaltose in patients with chronic kidney disease: A single center study. Indian J. Nephrol..

[16] Boots J.M.M., Quax R.A.M. (2022). High-Dose Intravenous Iron with Either Ferric Carboxymaltose or Ferric Derisomaltose: A Benefit-Risk Assessment. Drug Saf..

[17] Scott L.J. (2018). Ferric Carboxymaltose: A Review in Iron Deficiency. Drugs.

[18] Schaefer B., Tobiasch M., Viveiros A., Tilg H., Kennedy N.A., Wolf M., Zoller H. (2021). Hypophosphataemia after treatment of iron deficiency with intravenous ferric carboxymaltose or iron isomaltoside—A systematic review and meta-analysis. Br. J. Clin. Pharmacol..

[19] Kim S.K., Seo W.Y., Kim H.J., Yoo J.J. (2018). Postoperative Intravenous Ferric Carboxymaltose Reduces Transfusion Amounts after Orthopedic Hip Surgery. Clin. Orthop. Surg..

[20] Schaefer B., Zoller H., Wolf M. (2022). Risk Factors for and Effects of Persistent and Severe Hypophosphatemia Following Ferric Carboxymaltose. J. Clin. Endocrinol. Metab..

[21] Bernabeu-Wittel M., Aparicio R., Romero M., Murcia-Zaragoza J., Monte-Secades R., Rosso C., Montero A., Ruiz-Cantero A., Melero-Bascones M. (2012). PAHFRAC-01 investigators. Ferric carboxymaltose with or without erythropoietin for the prevention of red-cell transfusions in the perioperative period of osteoporotic hip fractures: A randomized contolled trial. The PAHFRAC-01 project. BMC Musculoskelet. Disord..

[22] Klein K., Asaad S., Econs M., Rubin J.E. (2018). Severe FGF23-based hypophosphataemic osteomalacia due to ferric carboxymaltose administration. BMJ Case Rep..

